# Nerve Instruments

**Published:** 1863-12

**Authors:** 


### NERVE INSTRUMENTS.

We have, for some four months, been using a set of nerve instruments, the same as those exhibited by Dr. Palmer, at the American Dental Association; and find them superior to anything we have before used.

The little "pods" for enlarging and clearing out nerve canals, are very excellent; indeed, we can scarcely conceive of any form of instrument that would accomplish the work with such facility and ease.

The plugging instruments, too, work nicely; and altogether they make by far the most complete set of nerve instruments ever presented to the profession. A description and drawings of them are given in the Transactions of the American Dental Association, to which we would refer our readers; or perhaps it would be better to refer them to Mr. S. S. White of Philadelphia, or Mr. Sherwood of this city,—both of whom are manufacturing these instruments very perfectly.

No operator, who is up with the times, can afford to be without them.

The whole set consists of fifteen to eighteen instruments.

